# Personal Rights and Preventive Medicine

**Published:** 1909-04-17

**Authors:** 


The Hospital

A Journal of

The Medical Sciences and Hospital Administration.

New Series. No. 111, Vol. V. [No. 1183, Vol. XLVI.] SATURDAY, APRIL 17, 1909.

### Personal Rights and Preventive Medicine.

A tract has been forwarded to our offices from the Personal Rights Association which contains certain letters from the Committee of that organisation to the War Office and Admiralty, with the replies; and as the matter discussed has bearings which affect much wider issues than those immediately under consideration, it may be not amiss, even at the risk of repeating an oft-told tale, briefly to examine some of them. The particular point which aroused the anxiety of the Association was a hope expressed publicly by a naval surgeon at the last annual meeting of the British Medical Association that at some future date the progress of bacteriological research will render possible efficient vaccination against syphilis, and that such vaccination will be carried out as a routine in the same way as is the prevention of smallpox. This expression of individual opinion seems to have been magnified by those for whom the author of the pamphlet under consideration acts as spokesman into the semblance of a very grave and imminent attack upon the liberty of the subject; and in consequence the Government departments were bombarded with indignant letters and repeated questions in Parliament. We regret that the sensible attitude maintained at first by the heads of the War Office and Admiralty was, after persistent browbeating, abandoned somewhat by the First Lord of the latter.

Now on the question of the preventive treatment of venereal disease, whether by vaccines or mercurial ointments or any other methods, we do not propose on this occasion to dilate, further than to point out that the great bulk of medical and scientific opinion is opposed to the views held by those who secured the repeal of the Contagious Diseases Acts, and that there are large numbers of earnest and eminent men and women of all parties who agree with the profession in the matter. There is much to be said over such a question, but as the Association reverted from the particular to the general in the various memorials which were forwarded to the Secretaries of State, it will be well to follow their example. It is stated that the services of the Army and Navy are unpopular among large classes of the people owing to the "tyrannical subversion of these liberties" (of the country) "by medical faddists and experimenters and others," and also that there is greater jeopardy to those who do enter the services from the injection "of vaccines, serums, and antitoxins from the witches' cauldron of the latest craze in therapeutics" than from foreign foes. Anything more utterly opposed to the truth we do not remember to have recently read. The absurdity of these and other similar statements might be left to the common sense of the public were it not that similar innuendoes have been bandied across the floor of the House of Commons, under the guise of questions to Ministers, and are now being distributed broadcast to those who may not understand that such a proceeding is no guarantee of their truth.

The situation resolves itself, therefore, into this—that the Personal Rights Association objects to all forms of preventive treatment (and presumably of curative treatment also) which involve the use of bacteria or bacterial products or the applications of bacteriology. By a convenient fiction they speak of vaccination *against* syphilis as vaccination *with* syphilis, and affect to treat the operation as the actual inoculation of the patient with that disease. But they go much further than this, for the passages we have quoted can logically bear only the wider interpretation we have just given them. If these principles are pushed to their natural conclusion the garrison of Malta must once more be fed on goats' milk, and Malta fever must continue to decimate the unfortunate troops and sailors; for it was the "medical faddists" who by their "trial of experiments" established the ætiology of the disease, and by their "shameless communication" of it to "the animals which are, perhaps, the nearest relations of man" abolished it and the enormous annual suffering which it entailed. So, too, on the same lines the campaigns against malaria, sleeping sickness, yellow fever, and dozens of other tropical scourges must be abandoned. After some laudation of themselves as "patriotic citizens of a nation which has won its renown and strength in the past by its devotion to the principles of freedom," the Committee later on put up one of the Vice-Presidents to ask in Parliament whether the First Lord of the Admiralty would give an assurance that no man on entering the Navy should be subjected to voluntary or compulsory syphilisation by the surgeons or others in authority. Passing by the equivocation, deliberate though it no doubt was, which is conveyed by the term syphilisation, we would emphasise the three preceding words as evidence of what the Personal Rights Association means by the principles of freedom. We would further call to the attention of this Association that the phrase "blood poisoning," when employed as equivalent to and synonymous with vaccine and serum and antitoxin treatment, is a gross misuse of language, the effect of which must be to deceive the uninstructed. Otherwise several hundred thousand—perhaps several million—children are being saved from death annually by "blood poisoning" with diphtheria antitoxin; and thousands are willingly undergoing "blood poisoning" at their own request at this moment for various diseases. If to inject substances under the skin is to poison the blood, then morphia and strychnine thus given are every whit as much to be prohibited as vaccines or any other preparation of dead microbes or microbic products. Even the Personal Rights Association might hesitate to describe the injection of strychnine as blood poisoning; yet we do seriously call to their notice the strict analogy between such a drug and tuberculin, or any other non-living therapeutic agent. Both are the products of vegetable life, both are dead, neither is injected into the blood; what essential difference is there between them?

